# Tet Enzymes, Variants, and Differential Effects on Function

**DOI:** 10.3389/fcell.2018.00022

**Published:** 2018-03-05

**Authors:** Philippa Melamed, Yahav Yosefzon, Cfir David, Anna Tsukerman, Lilach Pnueli

**Affiliations:** Faculty of Biology, Technion-Israel Institute of Technology, Haifa, Israel

**Keywords:** Tet enzymes, DNA methylation, hydroxymethylation, isoform, CXXC, 5mC, 5hmC

## Abstract

Discovery of the ten-eleven translocation 1 (TET) methylcytosine dioxygenase family of enzymes, nearly 10 years ago, heralded a major breakthrough in understanding the epigenetic modifications of DNA. Initially described as catalyzing the oxidation of methyl cytosine (5mC) to hydroxymethyl cytosine (5hmC), it is now clear that these enzymes can also catalyze additional reactions leading to active DNA demethylation. The association of TET enzymes, as well as the 5hmC, with active regulatory regions of the genome has been studied extensively in embryonic stem cells, although these enzymes are expressed widely also in differentiated tissues. However, TET1 and TET3 are found as various isoforms, as a result of utilizing alternative regulatory regions in distinct tissues. Some of these isoforms, like TET2, lack the CXXC domain which probably has major implications on their recruitment to specific *loci* in the genome, while in certain contexts TET1 is seen paradoxically to repress transcription. In this review we bring together these novel aspects of the differential regulation of these Tet isoforms and the likely consequences on their activity.

## The TET enzymes, DNA hydroxymethylation, and demethylation

The family of ten-eleven translocation (TET) methylcytosine dioxygenases are considered to play a major role in maintaining the fidelity of DNA methylation patterns through mediating demethylation (reviewed by Wu and Zhang, 2017). The three family members catalyze the hydroxylation of DNA methyl cytosine (5mC) into 5-hydroxymethylcytosine (5hmC), and can further oxidize 5hmC to 5-formylcytosine (5fC) and 5-carboxycytosine (5caC). During active demethylation, these 5fC- and 5caC-modified cytosines are rapidly excised by thymine DNA glycosylase (TDG), after which they are replaced by unmodified cytosines through base excision repair (BER) mechanisms (Tahiliani et al., 2009; Ito et al., 2010, 2011; He et al., 2011; Maiti and Drohat, 2011; Schomacher and Niehrs, 2017).

However, 5hmC is readily detected in many cell types, particularly in embryonic stem cells (ESCs) in which its levels decrease during differentiation (Globisch et al., 2010; Szwagierczak et al., 2010; Koh et al., 2011; Wu et al., 2011a). It is also found at notably high levels in adult neuronal cells (15–40% of 5mC and 1% of all cytosine in human brain), mostly in transcribed sequences, and in regions near promoters and enhancers, where it correlates positively with gene expression (Kriaucionis and Heintz, 2009; Münzel et al., 2011; Szulwach et al., 2011b; Colquitt et al., 2013; Hahn et al., 2013). This suggests that, at least in some tissues, 5hmC has a low turnover and thus, besides acting as intermediate of active demethylation, it functions as an epigenetic mark. In this context, 5hmC probably alters the local chromatin environment through the recruitment or displacement of proteins: a large number of proteins bind selectively to 5hmC-modified DNA (Spruijt et al., 2013) and some 5mC binding proteins do not recognize 5hmC (Hashimoto et al., 2012; Mellén et al., 2017). Furthermore, DNMT1, which is recruited to hemimethylated CpG during DNA replication, shows lower activity at sites of hemi-5hmC, which would also facilitate DNA demethylation in replicating cells (Valinluck and Sowers, 2007).

Many CpG-rich promoters contain 5hmC around 500–2,000 bases upstream and downstream of the transcription start site (TSS), but not at the TSS itself, where there is typically almost no modified cytosine (Szulwach et al., 2011a). Levels of 5hmC are characteristically highly enriched also at active or poised distal regulatory elements, as well as within gene bodies, and these serve as a hallmark of active transcription. Moreover, all three TET proteins are found strongly enriched at gene promoters, especially those that are CpG-rich (Pastor et al., 2011; Stroud et al., 2011; Szulwach et al., 2011a; Wu et al., 2011b; Xu et al., 2011; Yu et al., 2012; Hahn et al., 2013).

The TET proteins, although all harboring the same catalytic activity, are involved in diverse biological processes likely, at least in part, due to their differential expression through development and in a cell-type specific manner (Ito et al., 2010; Koh et al., 2011). TET1 is highly and specifically expressed in ESCs, the inner cell mass of the blastocyst and in primordial germ cells (PGCs), but its expression is generally downregulated during differentiation. TET2 is also expressed in ESCs, and the catalytic activity of both enzymes is required for normal differentiation during ESC lineage specification (Koh et al., 2011). TET2 plays a unique role also in hematopoietic stem cell differentiation (Ko et al., 2010; Moran-Crusio et al., 2011) and *Tet2* mutations are associated with aberrant DNA methylation and myeloid malignancies (Delhommeau et al., 2009). TET3 is the only 5mC oxidase present immediately after fertilization, and it mediates the mass cytosine oxidation of the male pronucleus leading to global erasure of 5mC (Gu et al., 2011; Iqbal et al., 2011). Subsequently, both the 5mC on the maternal genome and the oxidized cytosines on the paternal genome are lost from the early embryo in a replication-dependent manner (Inoue and Zhang, 2011). After implantation, the inner cell mass undergoes genome-wide *de novo* DNA methylation at a time when TET1 and TET2 are highly expressed which may thus fine-tune the methylation patterns.

## Different mechanisms of regulating TET expression give rise to a variety of isoforms

**Figure 1:**
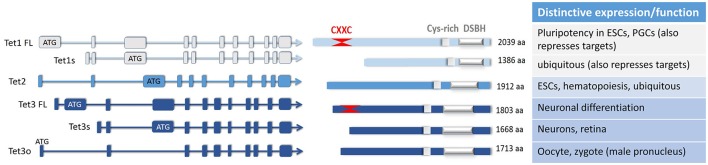
The major isoforms of the three TET enzymes, and their differential expression and or functions elucidated to date. A number of TET enzyme isoforms are produced as a result of differential promoter usage and/or alternative splicing, giving rise to distinct proteins of varied sizes (FL, full-length; s, short; o, ovarian isoform; aa, amino acids). All of these TET enzyme isoforms contain the C-terminal catalytic domain comprising the double-stranded β-helix domain (DSBH) which is adjacent to a Cys-rich region. As marked, one of the TET1 and one of the TET3 isoforms also have at their N-terminus two Cys4-type zinc finger motifs which make up the CXXC domain. Some of the distinctive or unique characteristics of these isoforms are noted; other details can be found in the main text.

A variety of regulatory distal enhancer and proximal promoter sequences appear to be utilized to direct expression of the TET enzymes in distinct contexts, and for TET1 and TET3 this gives rise to a number of transcripts encoding distinct proteins (Figure 1). The full-length canonical TET1 isoform appears virtually restricted to early embryos, ESCs and PGCs (Zhang et al., 2016), where it plays a crucial role in maintenance of pluripotency, its expression being driven by Oct3/4, Nanog, and Myc (Koh et al., 2011; Neri et al., 2015). However, even in the context of early embryonic development, two TSSs within a 15 kb-super-enhancer were shown to be utilized differently during the switch from naïve to primed pluripotent states, giving rise to *Tet1* transcripts with distinct 5′UTRs, although both encode the same protein. Expression of these transcripts is switched off with cell differentiation following DNA or histone (H3K27) methylation (Neri et al., 2015; Sohni et al., 2015; Zhang et al., 2016).

The dominant TET1 isoform in most somatic tissues, at least in the mouse, arises from alternative promoter usage which gives rise to a short transcript and truncated protein (Zhang et al., 2016; Good et al., 2017; Yosefzon et al., 2017). It is not clear what factors are responsible for the induction of this isoform in differentiated cells. However, it is evidently controlled by a distinct promoter which is driven by factors other than the pluripotency factors that activate the longer isoform, and ChIP-seq data indicates that multiple transcription factors bind this proximal regulatory region (Good et al., 2017). Transcriptional repression of *Tet1* expression has been described more extensively than its activation, and is implemented in partially-differentiated gonadotrope precursor cells by GnRH-induced activation of various kinase signaling pathways (Yosefzon et al., 2017), as well as by gonadal steroid hormones. Steroid regulation, especially by the liganded estrogen receptor (ESR1), is likely particularly crucial in reproductive-related cancers in which this TET1 isoform is activated aberrantly, being associated with worse outcome (Good et al., 2017). However, in some diseased states, the longer TET1 isoform is also expressed, regulated by additional mostly unidentified factors through the distal upstream promoter. This has been shown to include via activation of STAT3 and STAT5, which drive TET1 expression in AML, providing a possible target for treatment (Jiang et al., 2017). Expression of the short isoform is also regulated through a distal enhancer which encompasses one of the regions described as an alternative TSSs in ESCs (Sohni et al., 2015), and in the gonadotropes this region is protected from methylation by TET2 (Yosefzon et al., 2017).

Unlike TET1 and TET2, TET3 is not expressed in ESCs, but is the predominantly expressed TET enzymes in oocytes and zygotes, and is also expressed highly in neurons. In the oocytes, a unique TET3 isoform is found, its expression being initiated at a distinct promoter, presumably as a result of activation by oocyte specific factors (Jin et al., 2016). However, in neuronal cells it is transcribed to a number of different isoforms, as a result of alternative splicing and promoter usage, providing proteins with distinct N-termini (Liu et al., 2013). None of the exact mechanisms determining the cell-type specific expression of these isoforms has been fully elucidated, although total TET3 expression was increased during retinoic acid-induced differentiation of ESCs to the neuronal lineage (Jin et al., 2016).

TET2, although encoded by a single transcript, also utilizes alternative regulatory sequences in distinct contexts. Earlier studies showed that in mESCs its expression, like that of TET1, is regulated by Oct4 and Sox2 (Koh et al., 2011). More recently, *Tet2* was shown to contain a CpGI promoter with pluripotency-independent activity and an ESC-specific distal enhancer, which is down-regulated with differentiation to endow its distinct modes of regulation during development (Sohni et al., 2015). In more differentiated tissues, various cell-specific factors likely drive TET2 expression and in pre-B cells, the CCAAT/enhancer binding protein alpha (CEBPα) was seen to activate *Tet2* transcription which is necessary for the differentiation of these cells into functional macrophages (Kallin et al., 2012; Di Stefano et al., 2014).

There is evidence that the expression of all three TET enzymes can be regulated also post-transcriptionally by microRNAs (miRs). *Tet1* is down-regulated by miR29 family members, whose levels increase during differentiation of ESCs, and also in some cases of breast cancer correlating with increased tumor metastasis (Morita et al., 2013; Cui et al., 2016; Pei et al., 2016). *Tet2* expression was reported to be inhibited by more than 30 different miRs, and this was also shown to correlate with a drop in cellular 5hmC (Cheng et al., 2013). More specifically, miR22, which targets *Tet2*, is aberrantly upregulated in myeloid dysplastic syndrome and leukemia, and over expression of miR22 in mice models was seen associated with increased hematopoietic stem cell renewal and defective differentiation (Song et al., 2013). The repression of *Tet3* during neocortical development was seen to be regulated by miR15b, which is crucial in allowing the maintenance of the pool of neural progenitor cells (Lv et al., 2014). However, given that miRs characteristically target the 3′ end of the genes, and the various *Tet* isoforms differ primarily at their 5′ ends, it seems unlikely that regulation by these miRs plays a role in the differential targeting of the isoform expression.

TET activity is determined not only by the levels and type of transcript produced, but also to some degree by post-translational modifications. Ubiquitination, acetylation, phosphorylation, GlcNAcylation, and PARylation have all been reported to modify these enzymes, and would be expected to affect variously the protein localization, interacting partners and catalytic activity, as well as altering protein stability (reviewed by Wu and Zhang, 2017). In fact targeting of TET degradation by calpain, caspases, or targeting to the proteasome have been described as additional regulatory mechanisms (reviewed by Wu and Zhang, 2017), but the role and efficacy of these actions would obviously depend on which particular isoform is expressed.

## The inclusion or exclusion of the N-terminal CXXC domains and TET recruitment

It is still not clear how exactly the TET enzymes recognize their targets. The CXXC domains have been attributed a major role, as they are reported to bind unmethylated CpGIs (Xu et al., 2011), which would allow TET recruitment to these regions for protection of the DNA from aberrant methylation. However, the short TET1 isoform expressed in somatic tissues utilizes an initiating ATG in the third exon of the longer isoform, and encodes a truncated TET1 protein that completely lacks the CXXC domain. Although it does still bind DNA, the lack of this domain affects association of the protein with the DNA and binding affinity is reduced (Zhang et al., 2016). Notably however, the TET1 CXXC domain is a type three CXXC domain which was demonstrated to have little specificity for CpGs (Long et al., 2013), suggesting that its localization to CpGs, as well as that of the short isoform, is likely facilitated primarily by other proteins (Ko et al., 2013; Zhang et al., 2017). These mechanisms have largely yet to be described, but in the gonadotropes, both forms of TET1 affected *Lhb* gene expression similarly, although activity of the full-length TET1 was lost in the hypomethylated state, indicating distinct mechanisms of recruitment (Yosefzon et al., 2017). In contrast, overexpression of the short TET1 isoform in cancer cells had less effect on DNA methylation and gene expression than did the longer full-length form, and different genes were targeted. This was attributed to the distinct modes of their recruitment, as TET enzymes have been shown to interact with a large number of DNA-binding factors, some of which may recruit them to specific genomic *loci* (Zhang et al., 2016; Good et al., 2017; Wu and Zhang, 2017).

The CXXC domain of the full-length TET3, which is the predominant form expressed in neurons (Colquitt et al., 2013; Hahn et al., 2013), binds most strongly to CpGs modified by 5caC, although it does also bind unmethylated CpGs. This likely helps in its targeting to restricted locations in the genome, and it is found enriched on TSSs of a specific set of genes in the neuronal population (Jin et al., 2016). However, the other TET3 isoforms lack the CXXC domain. The first of these short forms is recruited to the DNA in the retina by the REST DNA-binding factor, which was seen to enhance TET activity on its target genes. This form of TET3 was also shown to interact with H3K36 methyl transferases which was suggested to further facilitate the active transcription of these genes (Perera et al., 2015). The short TET3 isoform, which lacks the CXXC domain, appears also to associate with a distinct CXXC4 protein that may direct its binding (Liu et al., 2013). For the oocyte-specific form, this aided recruitment was proposed to facilitate its genome-wide localization which would be important in the oxidation of the paternal genome immediately after fertilization (Jin et al., 2016). Mechanistically the reasons for this have yet to be elucidated given that both the longer CXXC-containing isoform and the CXXC4 domain protein appeared, at least *in vitro*, to have similar binding properties. However, it was hypothesized that association with distinct CXXC domains in other proteins might moderate TET function, as some CXXC domain proteins shown to interact with TET3 are involved with known signaling pathways such as Wnt/β-catenin (Liu et al., 2013).

The single TET2 isoform completely lacks a CXXC domain, and it has been suggested that the neighboring gene, which encodes a CXXC4 protein, IDAX, was originally part of an ancestral TET2 gene before chromosomal rearrangement during evolution (Ko et al., 2013). IDAX is now thought to play a role in regulating TET2 activity by facilitating its recruitment to unmethylated CpGs, although paradoxically it also down-regulates TET2 protein levels through caspase-mediated degradation (Ko et al., 2013). The levels and cellular localization of IDAX would thus play a role in targeting TET2 to specific sites as well as determining its activity levels.

## Different effects of different isoforms?

The distinct recruitment mechanisms of TET enzyme isoforms to modified or unmodified CpGIs, or CpGs in other contexts, would clearly play a role in determining their function. Notably only the global chromatin-binding, but not targeted binding of the short TET1 appears correlated with demethylation, and in mice expressing only the short isoform, PGC imprints were not erased and their progeny exhibited developmental defects (Zhang et al., 2016). Both findings imply not only distinct recruitment as described above, but also that the short isoform might function other than through its catalytic activity. In partially-differentiated gonadotrope precursors, the short TET1 isoform at the *Lhb* gene promoter is not catalytically active, and in other somatic tissues in which this is the dominant isoform, and in contrast to ESCs, TET1 colocalization with 5hmC is more rarely seen (Neri et al., 2013; Yosefzon et al., 2017). This may be due to the fact that the lacking N-terminus also contains another domain which enhances the catalytic activity of TET1 (Zhang et al., 2016). The fact that the aberrant elevated expression of this truncated TET1 isoform in cancers (breast, uterine, and ovarian) is associated with worse prognosis (Good et al., 2017) further indicates that the truncated isoform harbors distinct characteristics from those of the canonical full-length isoform, not only in the way it is recruited, but also in its function.

TET1 plays an important and very distinct role in regulating gene expression also, paradoxically, through repressing transcription, and its knockdown in mouse ESCs increased expression of many of its directly targeted genes (Williams et al., 2011; Wu et al., 2011b). A recent study on the role of TET1 in early stages of epiblast differentiation confirmed that the repression exerted on a subset of its target genes does not require TET1 catalytic activity, and is partially mediated *via* up-regulating the JMJD8 demethylase transcriptional repressor (Khoueiry et al., 2017). Studies on the gonadotrope precursors revealed that the short TET1 isoform represses the *Lhb* gene and its down-regulation is essential for this population of cells to complete differentiation. This effect is direct, and TET1 binds the upstream promoter at a region carrying H3K27me2/3 which was markedly reduced in cells in which TET1 was knocked down (Yosefzon et al., 2017). Given that many TET1-bound promoters are occupied by PRC2 and TET1 clearly plays a role in regulating PRC-targeted genes (Wu et al., 2011b), a role for TET1 in recruiting the EZH2 methyl transferase-containing PRC complex that targets H3K27 methylation seems likely. Notably, however, co-localization of PRC2 with 5hmC was apparent only in ESCs, implying that only the full-length form, which is expressed more highly the in these cells, is catalytically active in this complex (Neri et al., 2013).

TET1-mediated repression might also involve a role for the MBD3/NuRD repressor complex, which was shown in ESCs to co-localize with TET1, bind to 5hmC DNA with higher affinity than to 5mC, and its knockdown affected primarily expression of 5hmC-modified genes. Conversely, the knockdown of MBD3 also reduced levels of 5hmC, suggesting that it either plays a role in TET1 recruitment, or is recruited to 5hmC-modified DNA and plays another role in protecting them from complete demethylation (Yildirim et al., 2011). More recently it was shown that MBD1 enhances the activity of TET1 at heterochromatic DNA: the MBD1 CXXC domain that binds methylated CpGs helps recruit TET1 to the heterochromatin, but the resulting 5hmC then causes displacement of the MBD1 (Zhang et al., 2017). Other mechanisms of TET1-mediated repression have also been proposed, including its association with the Sin3A co-repressor complex, which has a similar binding profile and significant overlap of target genes. TET1 appears to facilitate Sin3A binding, though not *vice versa*, and after knock down of either factor, many genes bound by these factors had increased expression, indicating that Sin3A is required for a subset of TET1-repressed target genes (Williams et al., 2011). In ESCs, TET1 was seen associated with Sin3A at certain sites on bivalent genes which lack 5hmC, again revealing a lack of catalytic activity in this context (Neri et al., 2013).

The localization of the full-length TET3 near the TSS of promoters with unmethylated CpGIs implies its role in protection of these regions from methylation. Because this isoform binds with higher affinity to 5caC, if aberrant 5mC methylation does occur, the TET3-mediated oxidation to 5caC would ensure that binding of TET3 to this location is further strengthened. In neurons, genes found to be regulated in this way include those involved in controlling key genes of the BER pathway, suggesting a TET3-dependent regulatory feedforward pathway for active DNA demethylation. This would likely be particularly important in post-mitotic neurons in which replication-dependent passive demethylation of aberrant 5mC would not occur (Jin et al., 2016). Little is known about the differential effects of the shorter TET3 isoforms, although the ability of the neuronal isoform to interact with H3K36 methyl transferases was shown, and for at least one of them TET3 increased its enzymatic activity, providing a clear link between these two chromatin modifications associated with active transcription (Perera et al., 2015).

## Concluding remarks

Since the identification of the TET enzymes and their modification of the DNA, a vast amount of research has contributed to our understanding of this family of enzymes and of the role of 5hmC in the genomic landscape, particularly in the context of ESCs and early development. Notwithstanding, their abundant presence in the brain together with high levels of 5hmC, as well as the discovery of TET2's role in AML, prompted examination of other differentiated tissues, revealing the wide spread expression of these enzymes which work to regulate transcription through diverse mechanisms in distinct contexts. As emphasized here, some of this diversity undoubtedly arises from the differential regulatory mechanisms which drive expression of distinct isoforms. However, it remains far from clear how the functions of this group of enzymes developed and diverged to such a degree, and the role of their aberrant expression in disease.

## Author contributions

All authors listed have made a substantial, direct, and intellectual contribution to the work, and approved it for publication.

### Conflict of interest statement

The authors declare that the research was conducted in the absence of any commercial or financial relationships that could be construed as a potential conflict of interest.
